# Supplementary figures and images for: Neural responses to natural and model-matched stimuli reveal distinct computations in primary and nonprimary auditory cortex
Source: PLoS Biol. 2018 Dec 3;16(12):e2005127. doi: 10.1371/journal.pbio.2005127 (PMC6292651; doi:10.1371/journal.pbio.2005127)

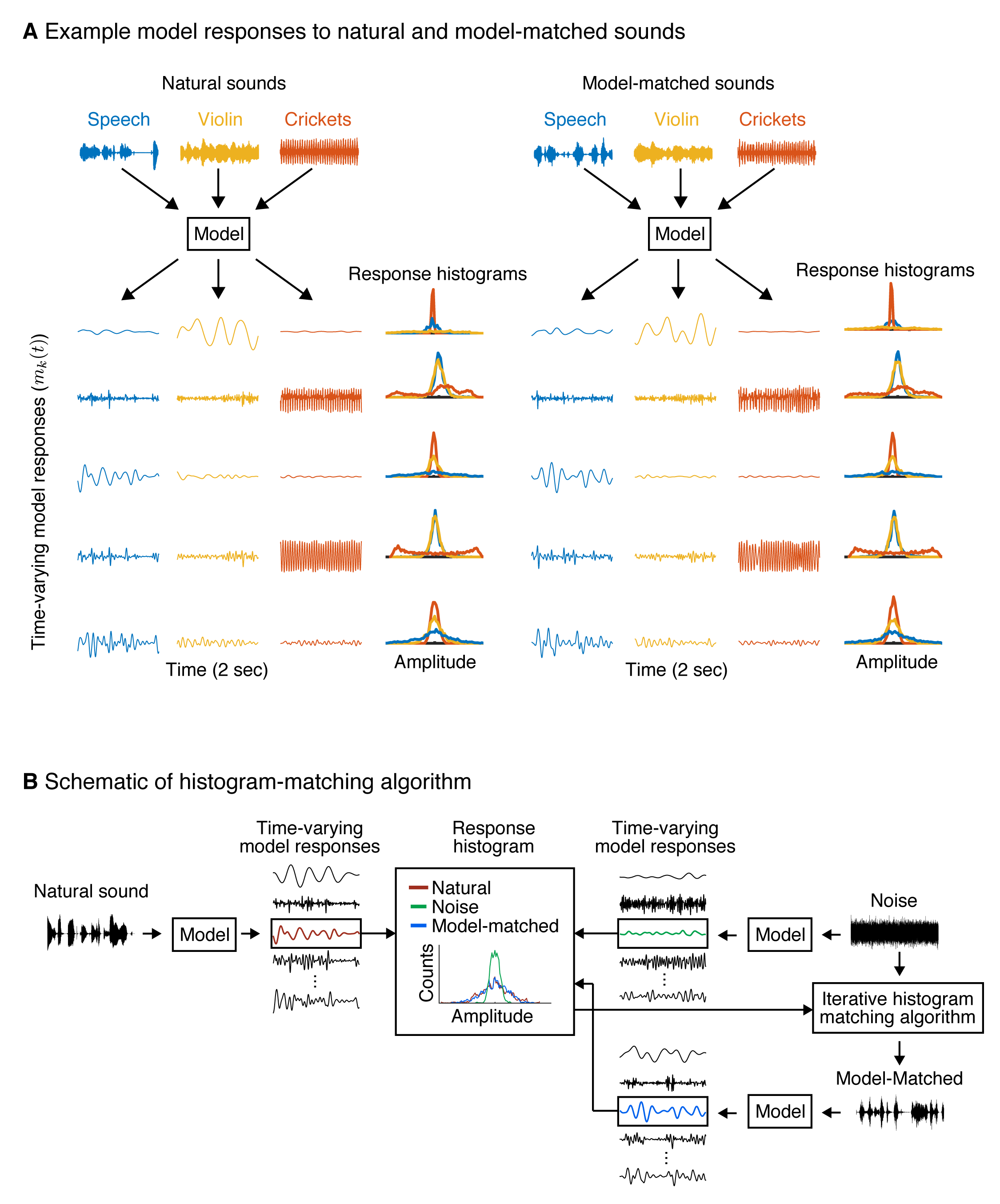

Supplement: S1 Fig — (A) The models considered here were defined by the response time course of a set of model features, each computed by filtering a cochleagram representation of sound (illustrated in Fig 1A). Our model-matching algorithm collapses these time courses across time to form a histogram and then generates a sound with the same histograms as a natural sound. Here, we plot example time courses and histograms for three example natural sounds (left panel) and corresponding model-matched sounds (right panel), in this case generated from the full spectrotemporal filter model used throughout this paper. Different natural sounds produce distinct response time courses and histograms. Corresponding natural and model-matched sounds produce similar response histograms but distinct response time courses. (B) The model-matched sounds were synthesized by modifying a noise signal so as to match the histogram of each feature’s response to a natural sound. The algorithm was initialized with Gaussian noise that was initially unstructured and thus produced feature responses with a different histogram and a different time-varying response pattern. The noise sound was then iteratively adjusted so as to match the histogram of each feature to the natural sound, while leaving the temporal pattern unconstrained. This figure plots histograms for one example model feature in response to a natural, noise, and model-matched sound. The histogram-matching algorithm is conceptually similar to a classic visual texture synthesis algorithm [48]. (TIF) [file pbio.2005127.s001.tif]

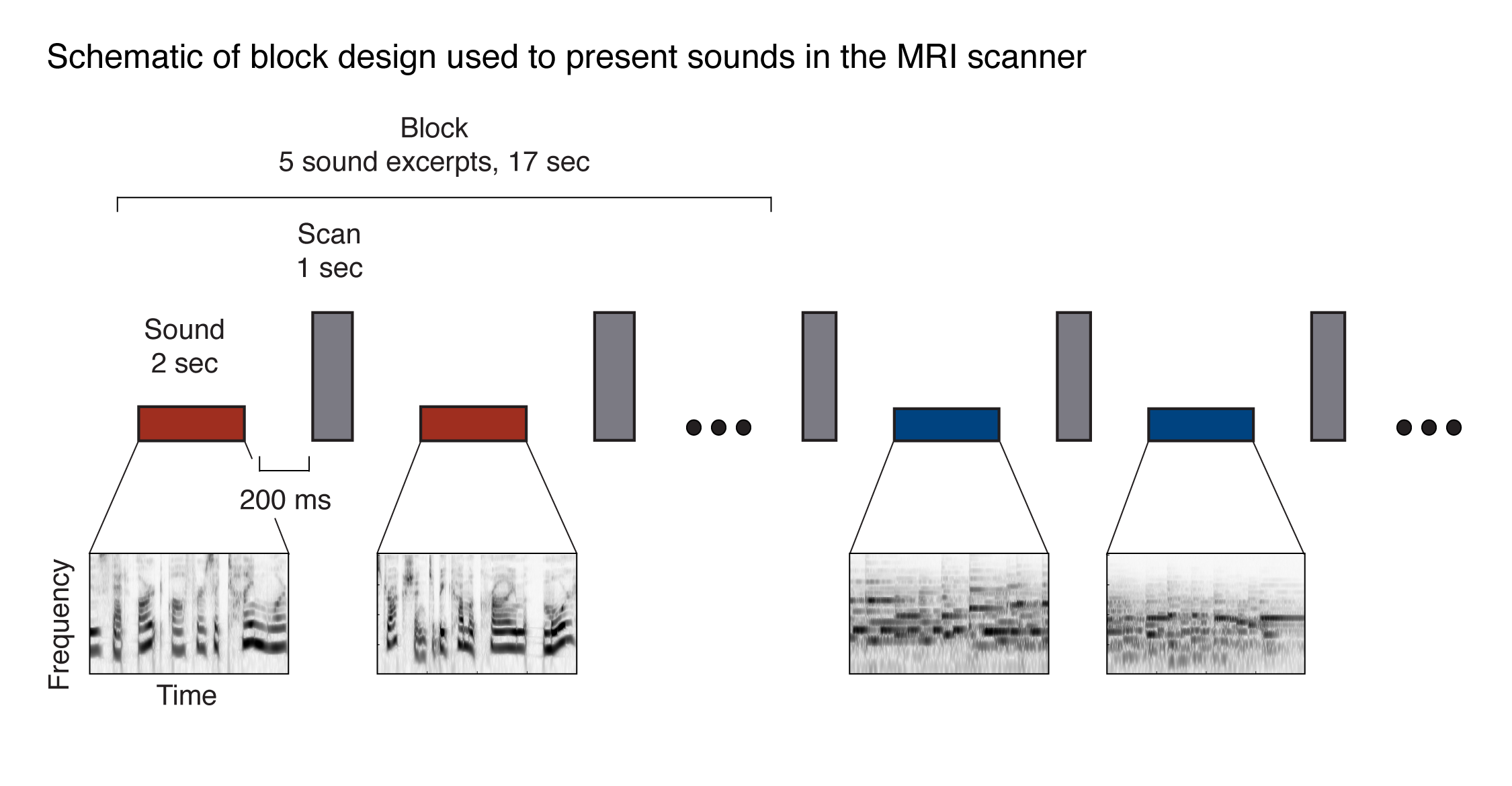

Supplement: S2 Fig — Each 10-second stimulus was subdivided into five 2-second segments. These five segments were presented in a random order, with a 1-second scan acquisition interspersed between each presentation (1.05 seconds for Paradigm II). A short 200-millisecond buffer was present between stimuli and scan acquisitions. The total duration of each “block” of five sounds was 17 seconds (17.25 seconds for Paradigm II). The response to a stimulus was computed as the average response of the second through fifth scan acquisition after block onset (the first acquisition was discarded to account for the hemodynamic lag). (TIF) [file pbio.2005127.s002.tif]

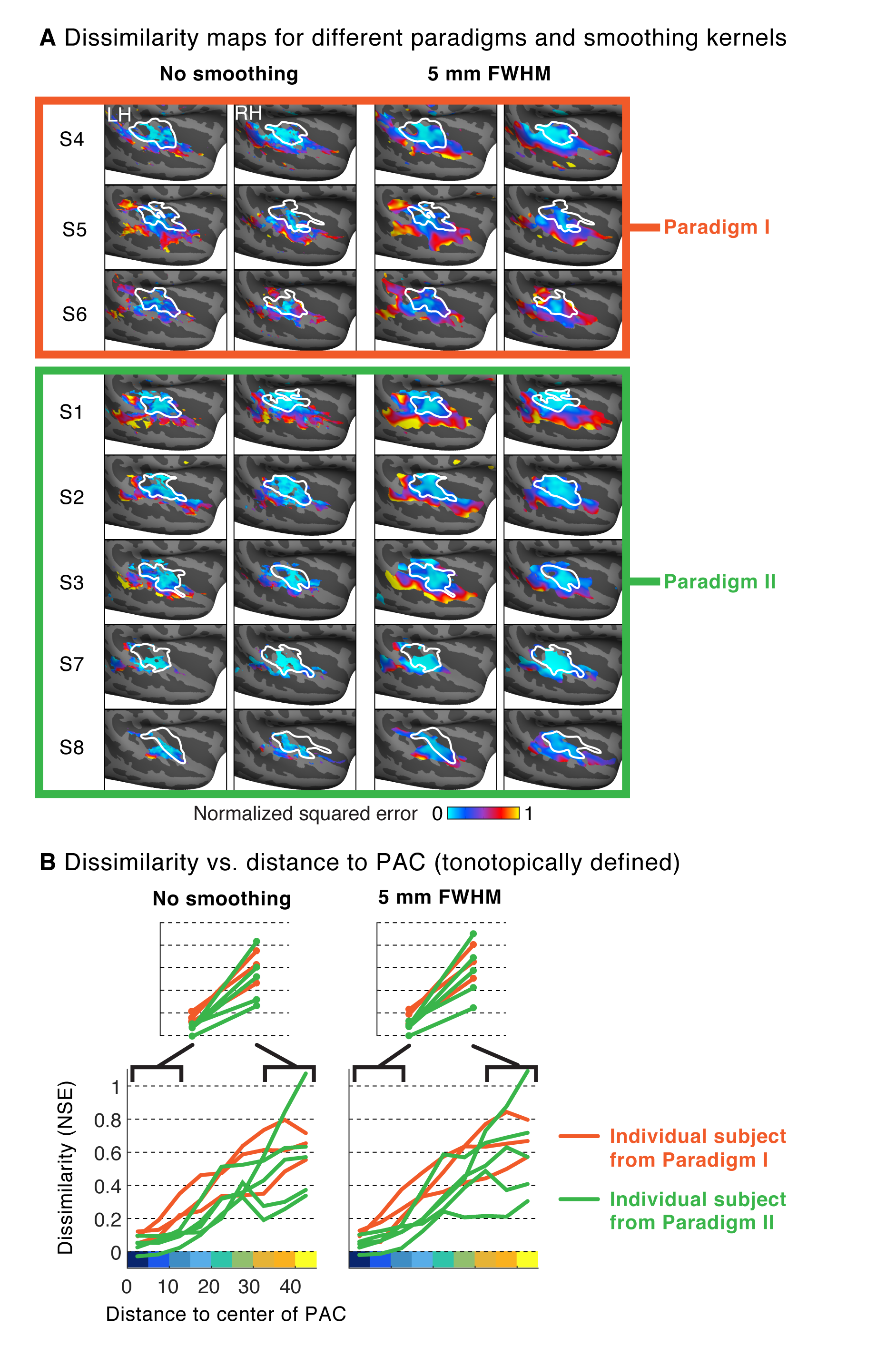

Supplement: S3 Fig — In Paradigm I, only the natural sounds were repeated. In Paradigm II, both natural and model-matched sounds were repeated. A smaller voxel size was employed in Paradigm II (2 mm isotropic instead of 2.1 × 2.1 × 4 mm for Paradigm I). (A) Natural versus model-matched dissimilarity maps computed with and without smoothing. Individual subjects are grouped by paradigm. Subjects are sorted by the reliability of their response to natural sounds for Paradigm I and by the reliability of their response to both natural and model-matched sounds for Paradigm II (measured using the NSE). (B) Annular analyses computed from data with and without smoothing. Each line corresponds to an individual subject and the color indicates the paradigm in which that subject was scanned (orange for Paradigm I and green for Paradigm II). NSE values are averaged across the left and right hemisphere because we observed similar trends in both hemispheres. NSE, normalized squared error. (TIF) [file pbio.2005127.s003.tif]

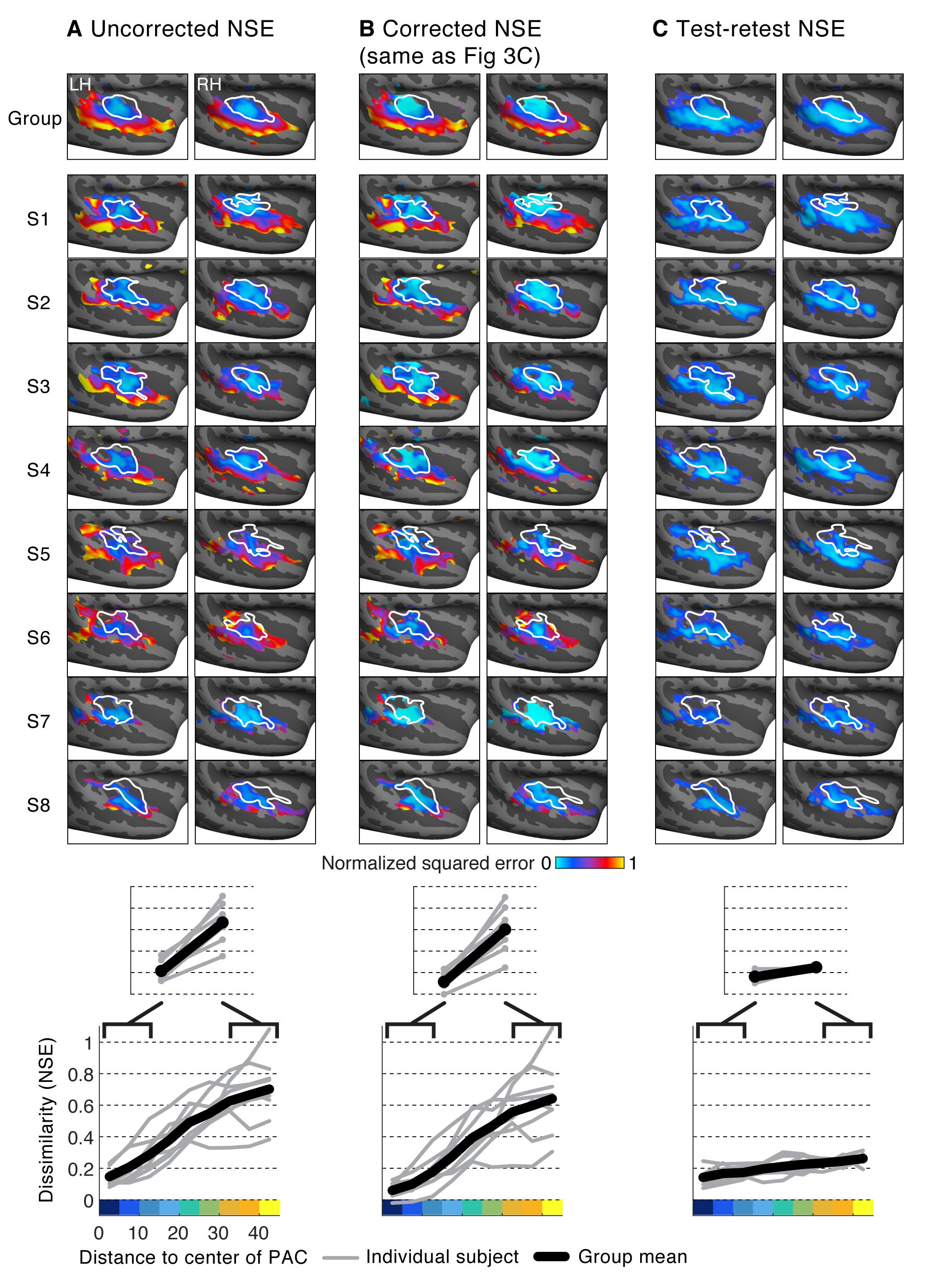

Supplement: S4 Fig — (A) The uncorrected NSE between responses to natural and model-matched sounds. (B) Corrected NSE maps (same as Fig 3C) replicated here for ease of comparison with the uncorrected maps. (C) Test-retest reliability of voxel responses measured with the NSE. Voxel reliability for Paradigm I (Group, S4, S5, and S6) is based on responses to natural sounds. Voxel reliability for Paradigm II (S1, S2, S3, S7, and S8) is based on responses to both natural and model-matched sounds (responses to natural and model-matched sounds were combined into a single vector, and we computed the NSE for multiple measurements of this vector). Distance-to-PAC analyses are shown at the bottom of each panel (PAC defined tonotopically). Format is the same as Fig 3C and 3D. NSE, normalized squared error; PAC, primary auditory cortex. (TIF) [file pbio.2005127.s004.tif]

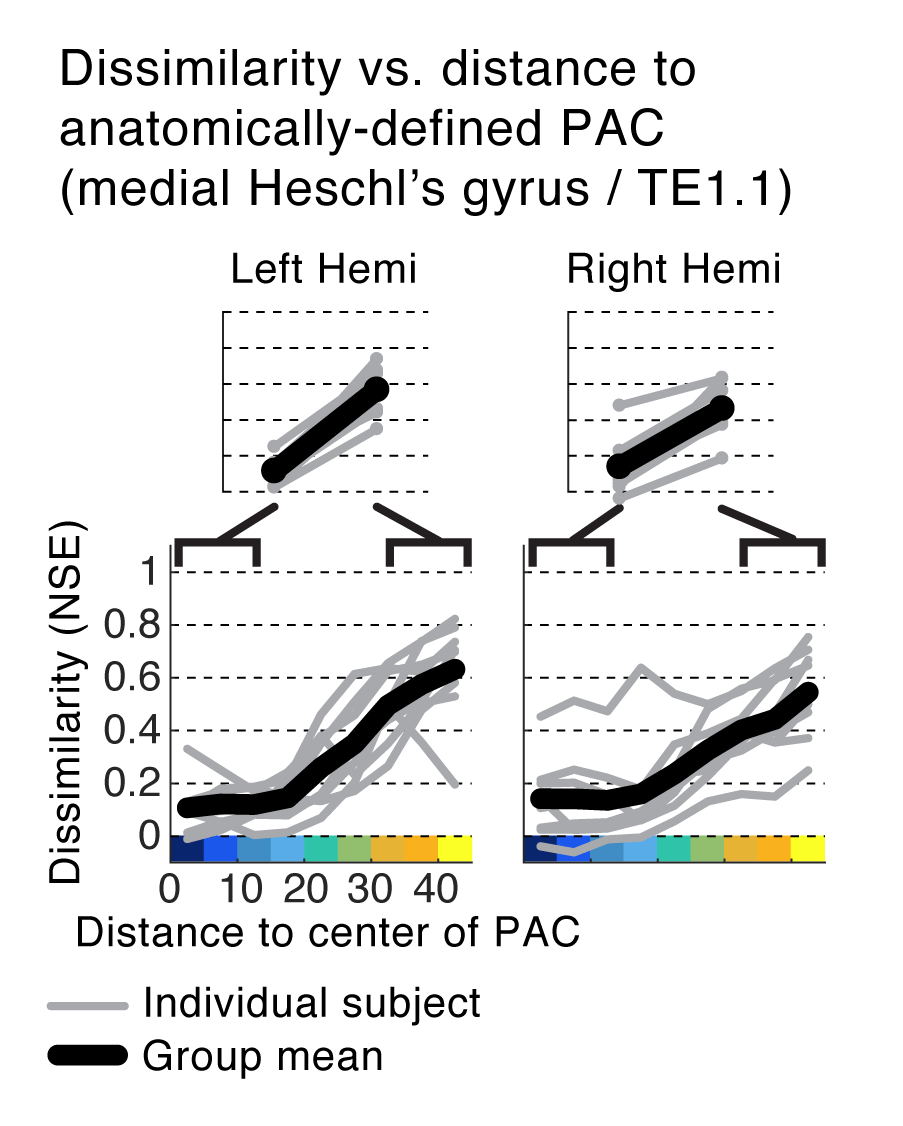

Supplement: S5 Fig — Voxels are binned based on their distance to the center of anatomical region TE1.1 [58], which is located in posteromedial Heschl’s gyrus. Format is the same as Fig 3D. PAC, primary auditory cortex. (TIF) [file pbio.2005127.s005.tif]

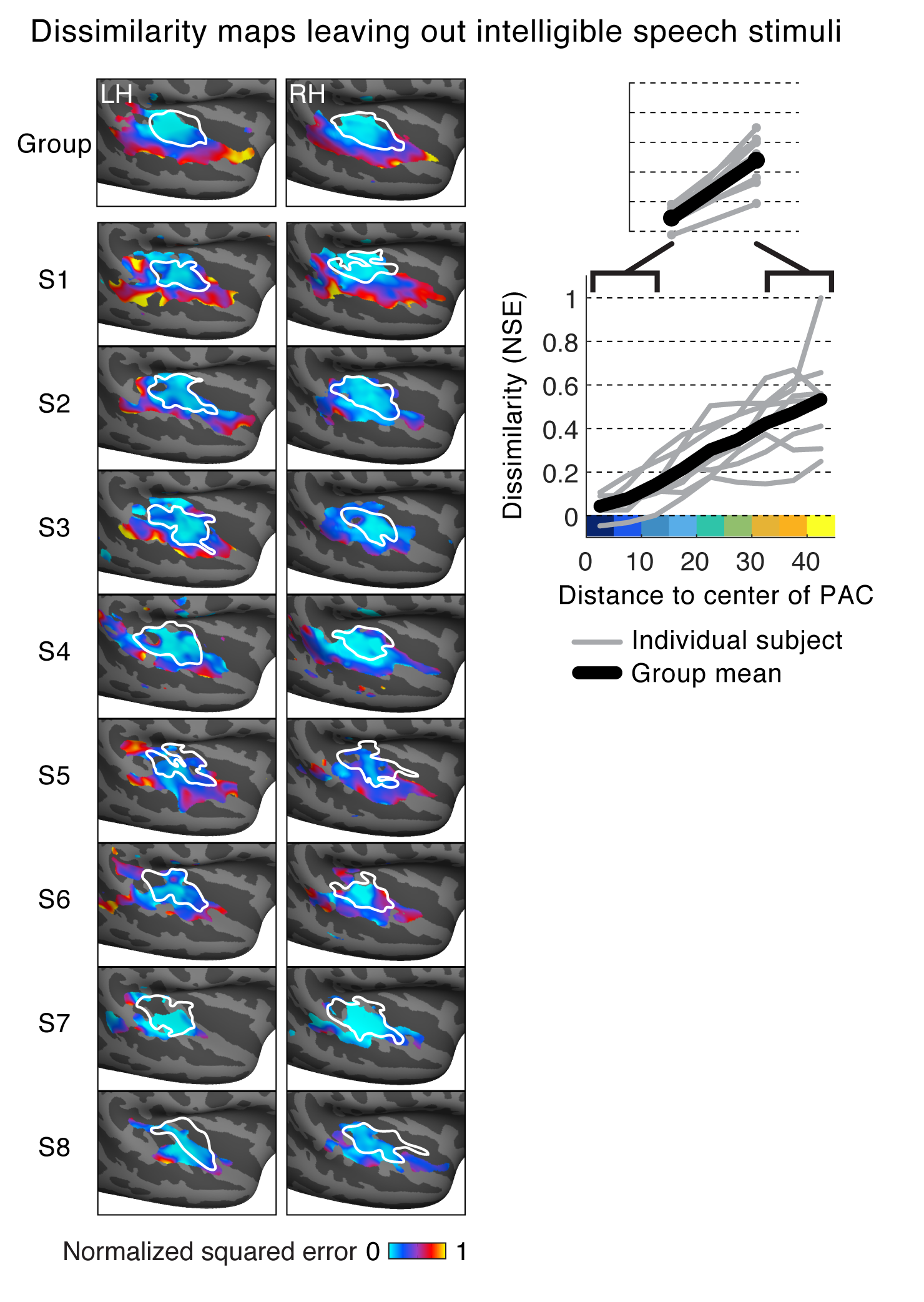

Supplement: S6 Fig — Maps plot the NSE between voxel responses to natural and model-matched sounds, omitting English speech and music with English vocals (all subjects were native English speakers). Format is the same as Fig 3C and 3D. NSE, normalized squared error. (TIF) [file pbio.2005127.s006.tif]

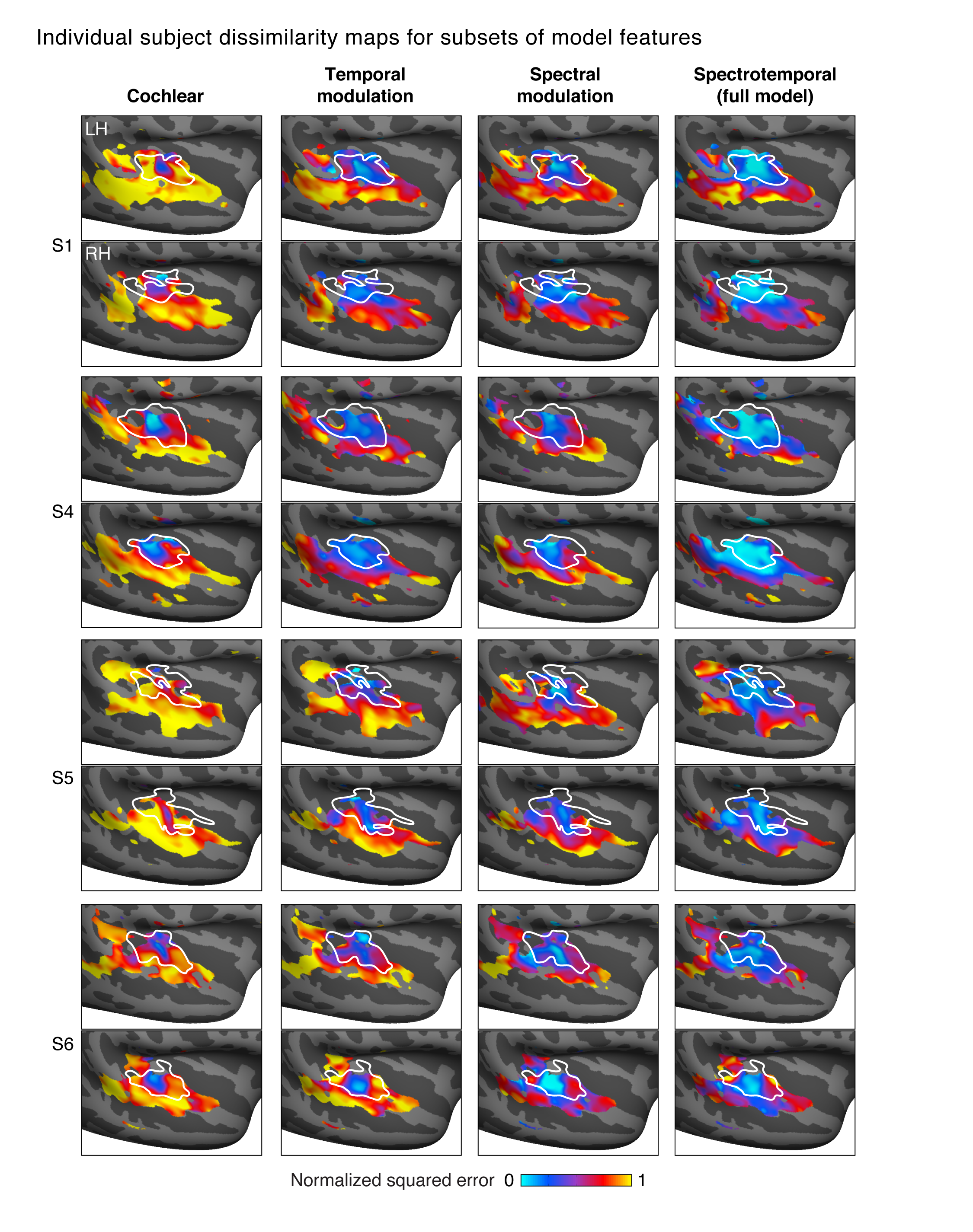

Supplement: S7 Fig — Format is the same as Fig 4B. Only subjects scanned in Paradigm I are shown, because Paradigm II did not include model-matched sounds constrained by subsets of model features. NSE, normalized squared error. (TIF) [file pbio.2005127.s007.tif]

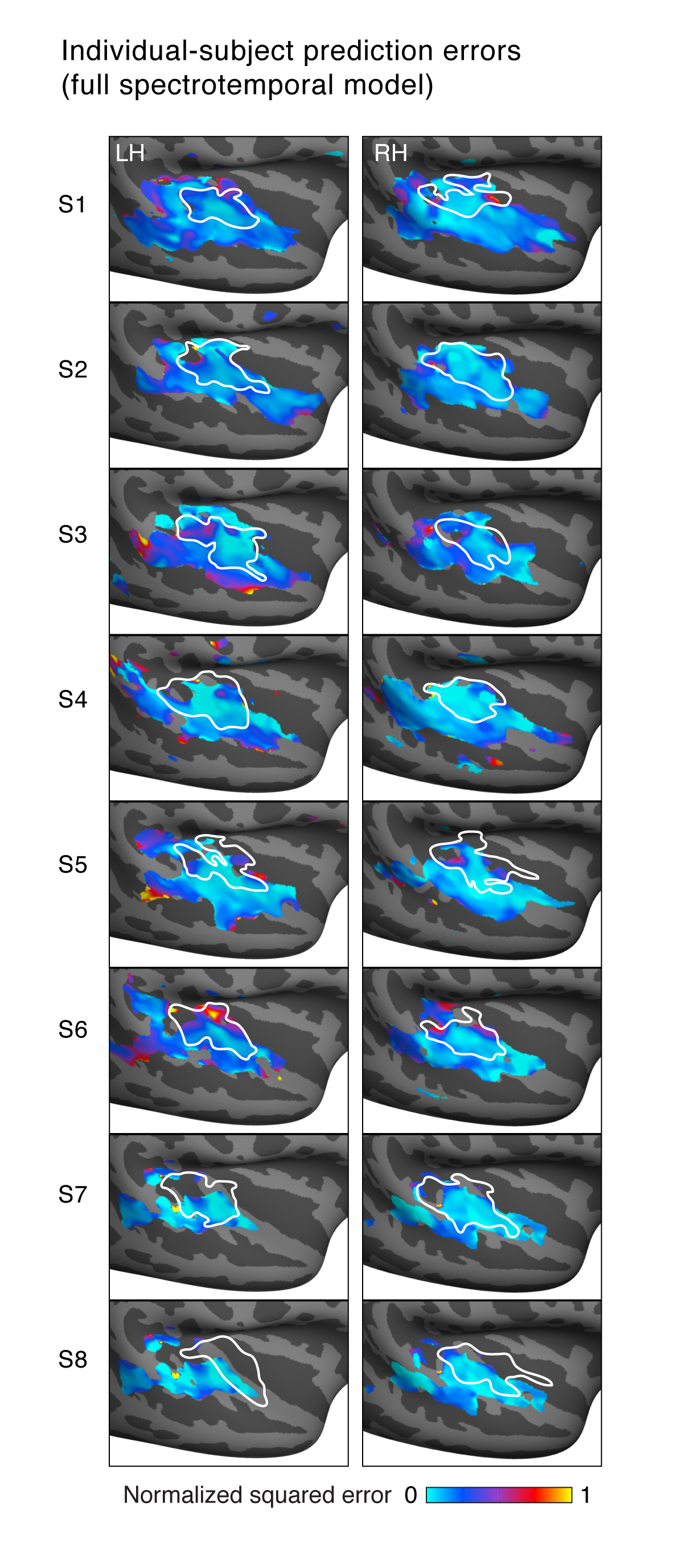

Supplement: S8 Fig — Format is similar to Fig 5B. (TIF) [file pbio.2005127.s008.tif]

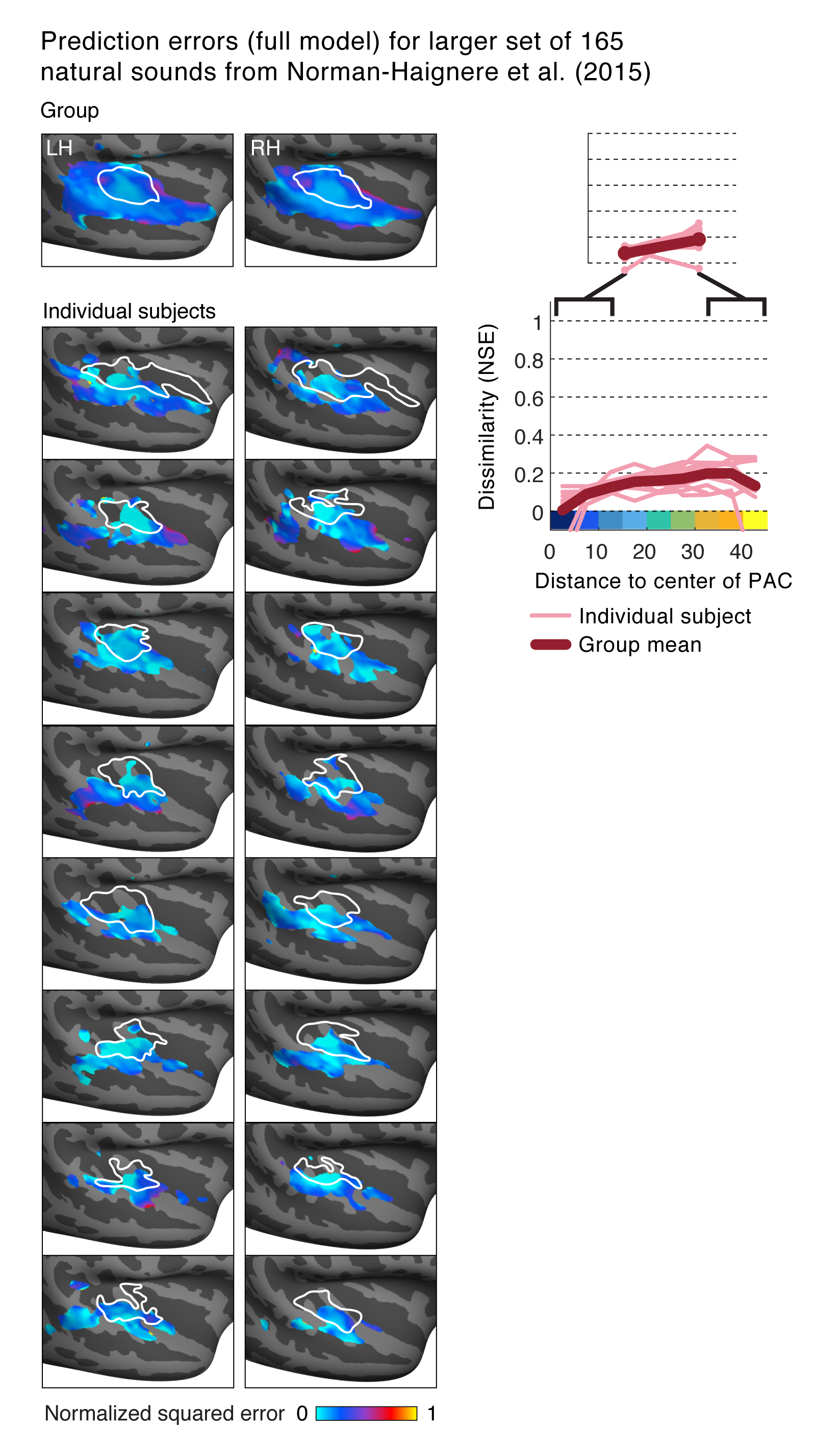

Supplement: S9 Fig — Format similar to Fig 5B and 5C. (TIF) [file pbio.2005127.s009.tif]

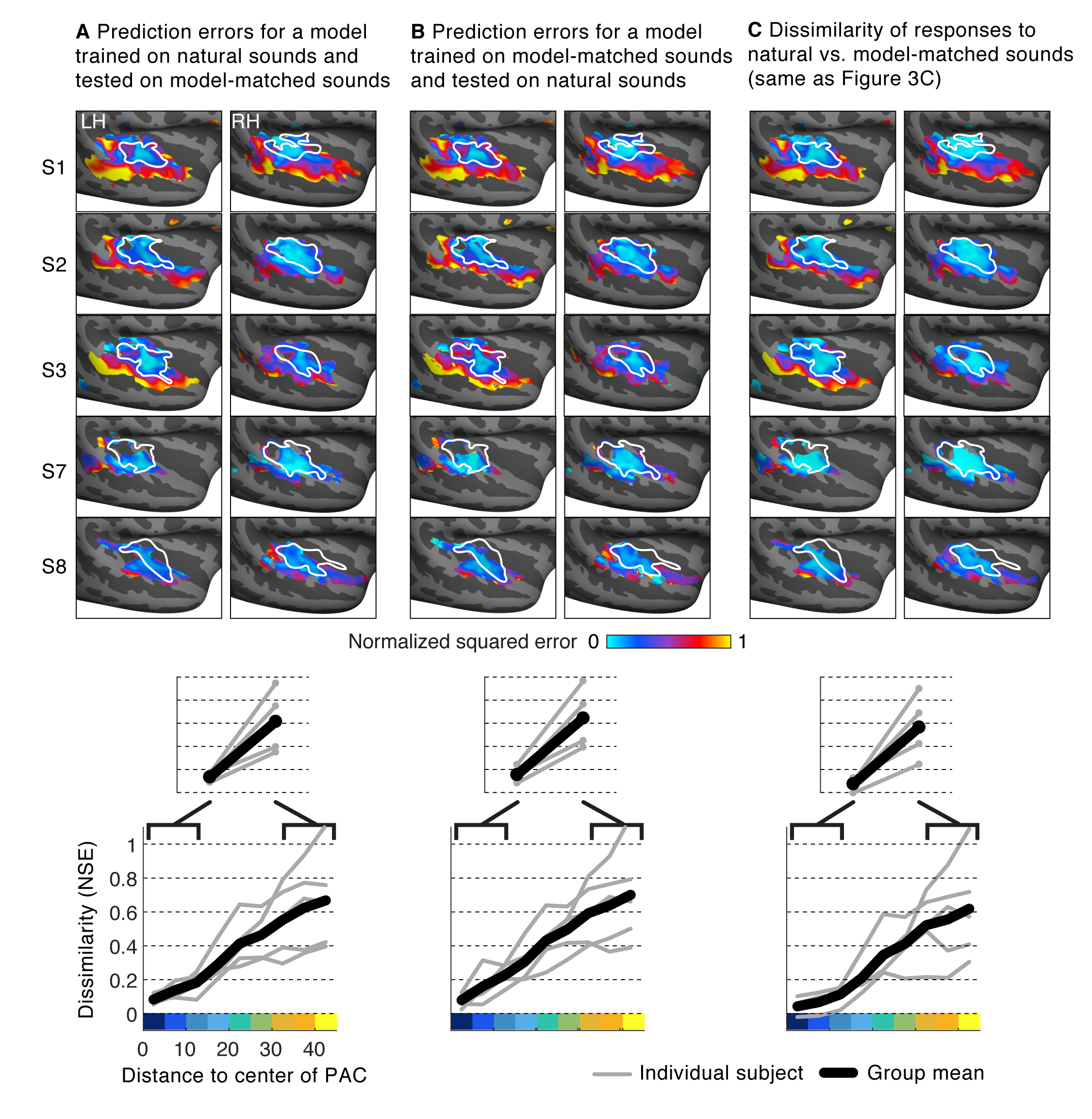

Supplement: S10 Fig — (A) Prediction error maps of a model trained on natural sounds and tested on model-matched sounds. (B) Prediction error maps of a model trained on model-matched sounds and tested on natural sounds. (C) For comparison, the error of the measured voxel response to natural and model-matched sounds is reproduced here (same as Fig 3C and 3D). Annular analyses summarizing the error as a function of distance to tonotopically defined PAC are shown below each set of maps. Data are shown for subjects scanned in Paradigm II, for whom both natural and model-matched sounds were repeated, which made it possible to noise-correct the predictions. PAC, primary auditory cortex. (TIF) [file pbio.2005127.s010.tif]

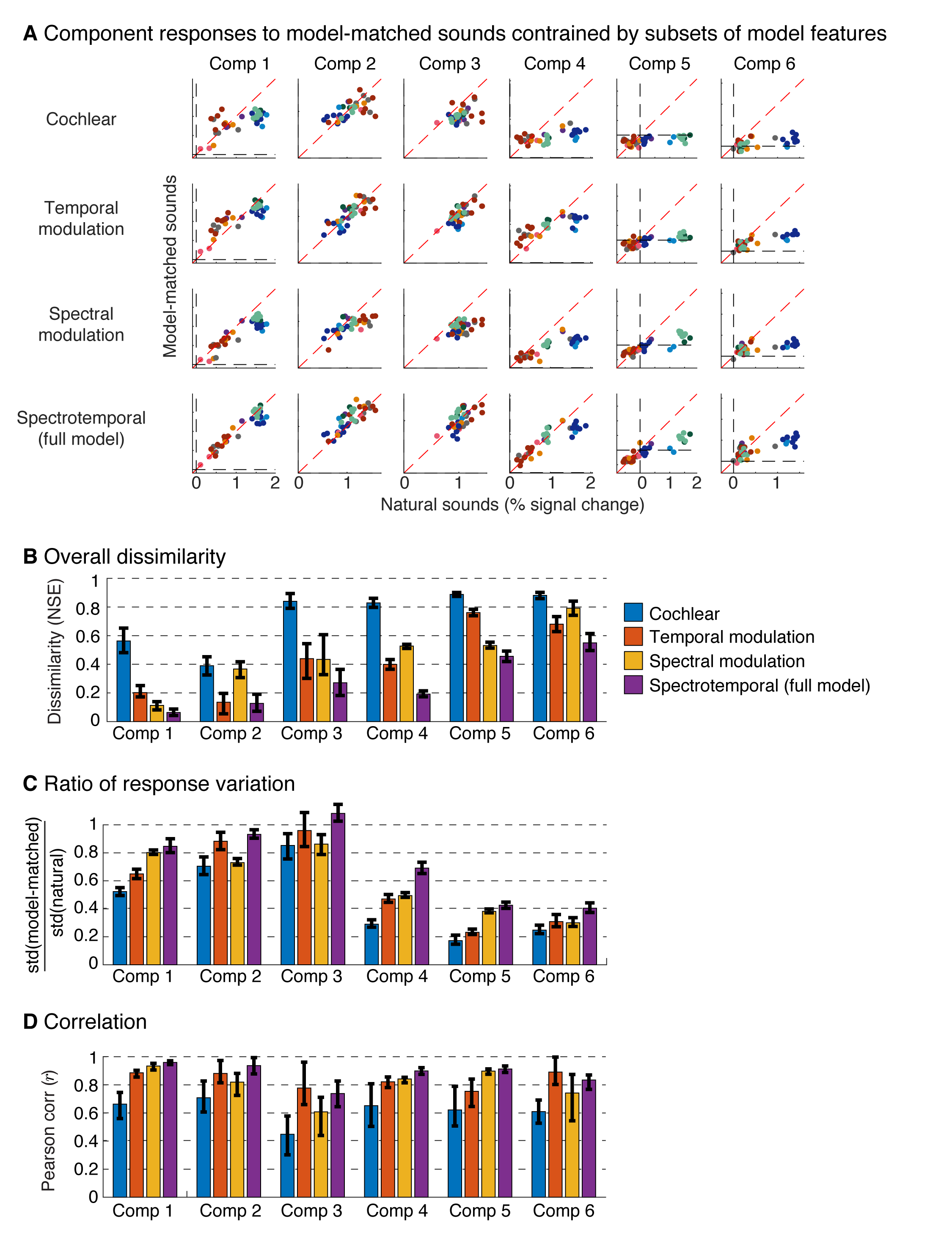

Supplement: S11 Fig — (A) Response of each component to each natural and corresponding model-matched sound. (B) NSE between natural and model-matched sounds. (C) Ratio of the standard deviation of responses to model-matched and natural sounds. (D) Correlation of responses to natural and model-matched sounds. NSE, normalized squared error. (TIF) [file pbio.2005127.s011.tif]

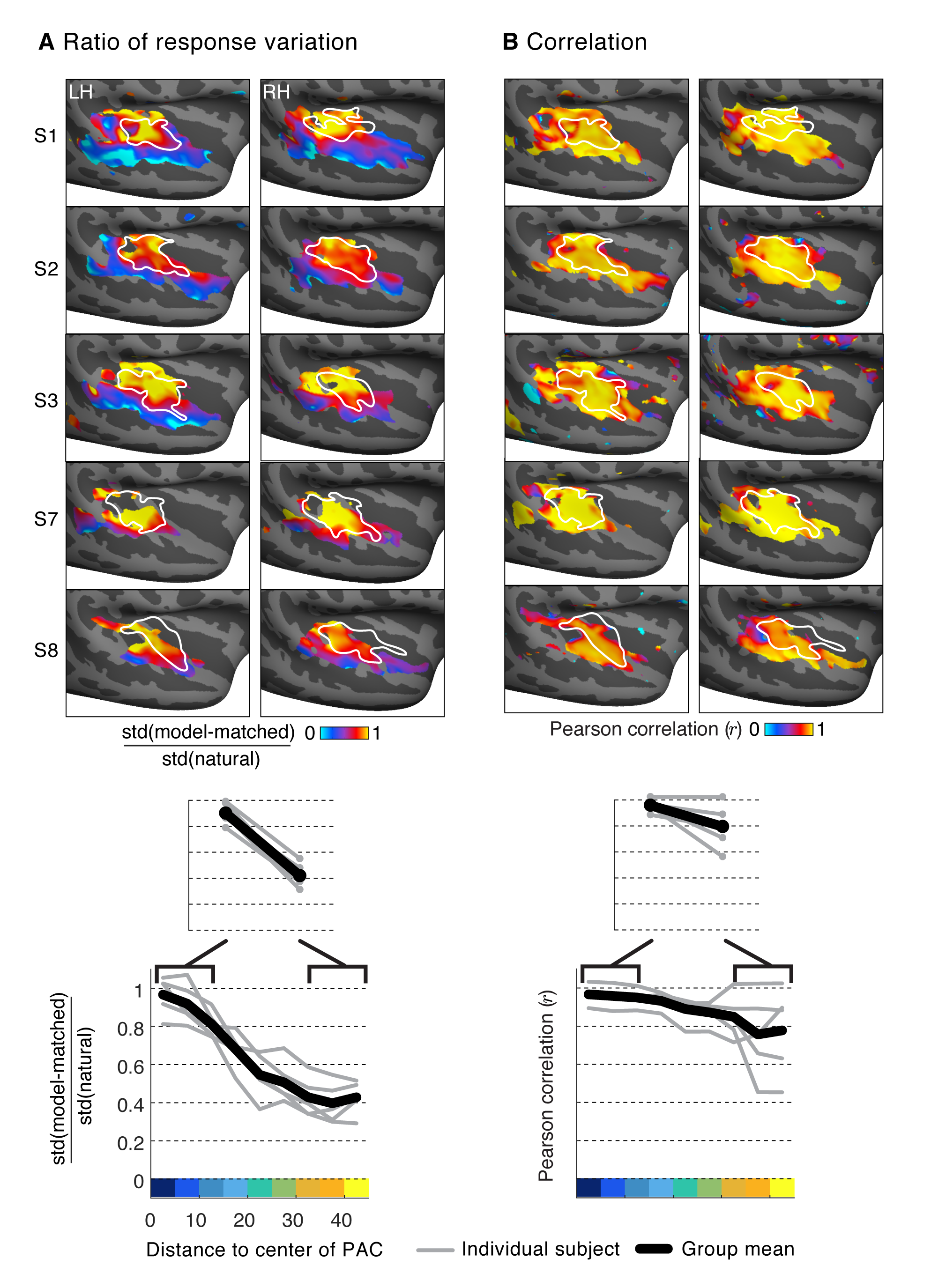

Supplement: S12 Fig — (A) Whole-brain maps plotting the variation in responses to natural versus model-matched sounds, measured as the ratio of the standard deviation of responses to the two sound sets. Cool colors indicated less response variation for model-matched sounds. Distance-to-PAC summary analysis is plotted below (PAC defined tonotopically). (B) Maps of the Pearson correlation between responses to natural and model-matched sounds, with distance-to-PAC analysis below. All of the measures have been corrected for noise. Analysis is based on data from Paradigm II, in which we measured responses to natural and model-matched sounds an equal number of times. For the response variation maps (panel A), we included all voxels with a reliable response across both natural and model-matched sounds (test-retest NSE < 0.4). For the correlation maps (panel B), we excluded voxels that did not have a reliable correlation to model-matched sounds (test-rest r < 0.4), as was the case in many nonprimary voxels, due to weak responses. For such voxels, it is difficult to estimate a reliable correlation, because the correlation is undefined as the variance of one variable goes to zero. NSE, normalized squared error; PAC, primary auditory cortex. (TIF) [file pbio.2005127.s012.tif]

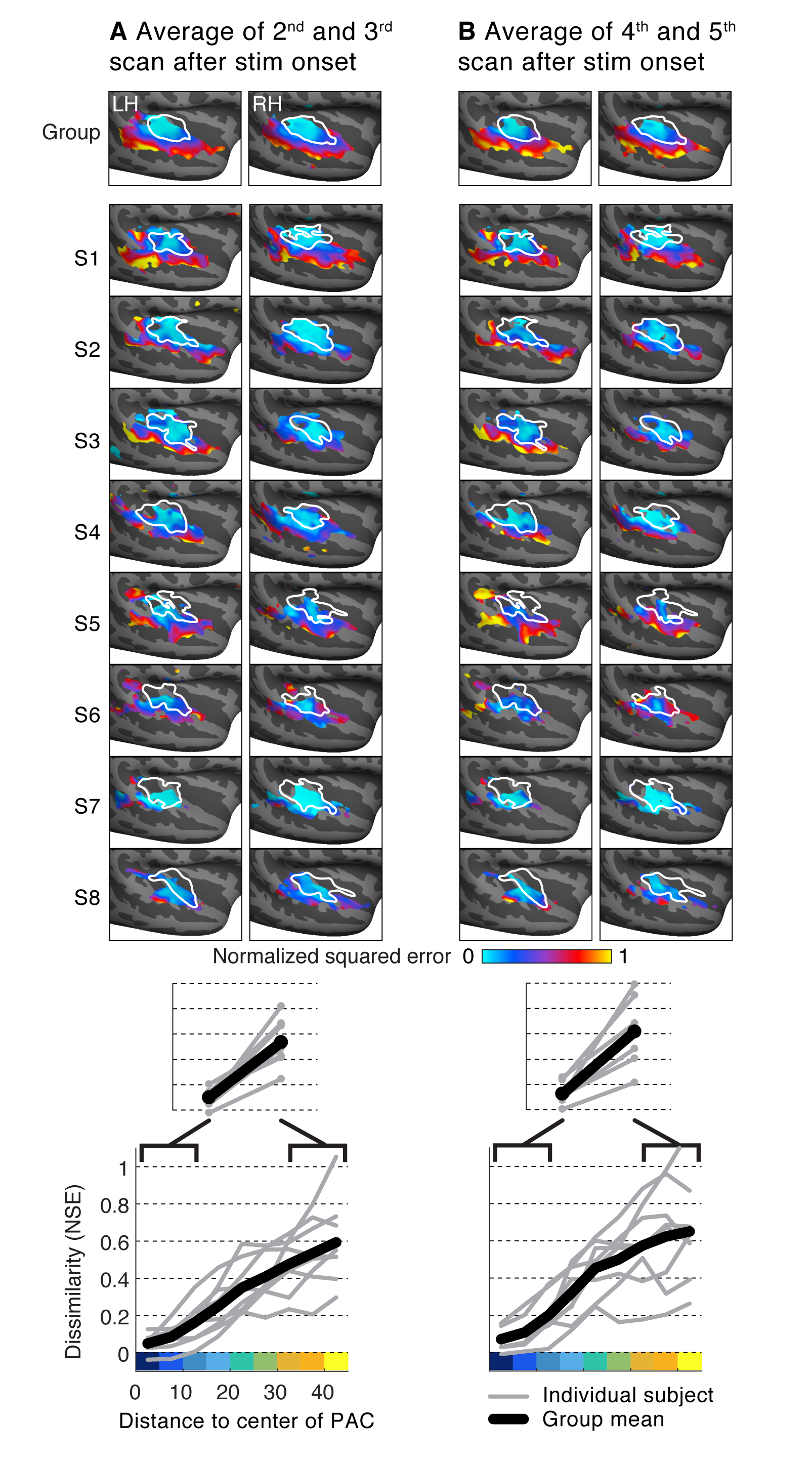

Supplement: S13 Fig — Each stimulus was 10 seconds in duration but was split up into five 2-second segments (see S2 Fig). After each segment, a single scan acquisition was collected. Analyses in the main text were based on the average response of the second through fifth acquisitions after the onset of each stimulus block (first acquisition was discarded to account for the hemodynamic delay). Here, we test the sensitivity of the results to the averaging window by restricting the analysis to data averaged across acquisitions 2 and 3 (panel A) or 4 and 5 (panel B). Compare with Fig 3C and 3D. (TIF) [file pbio.2005127.s013.tif]

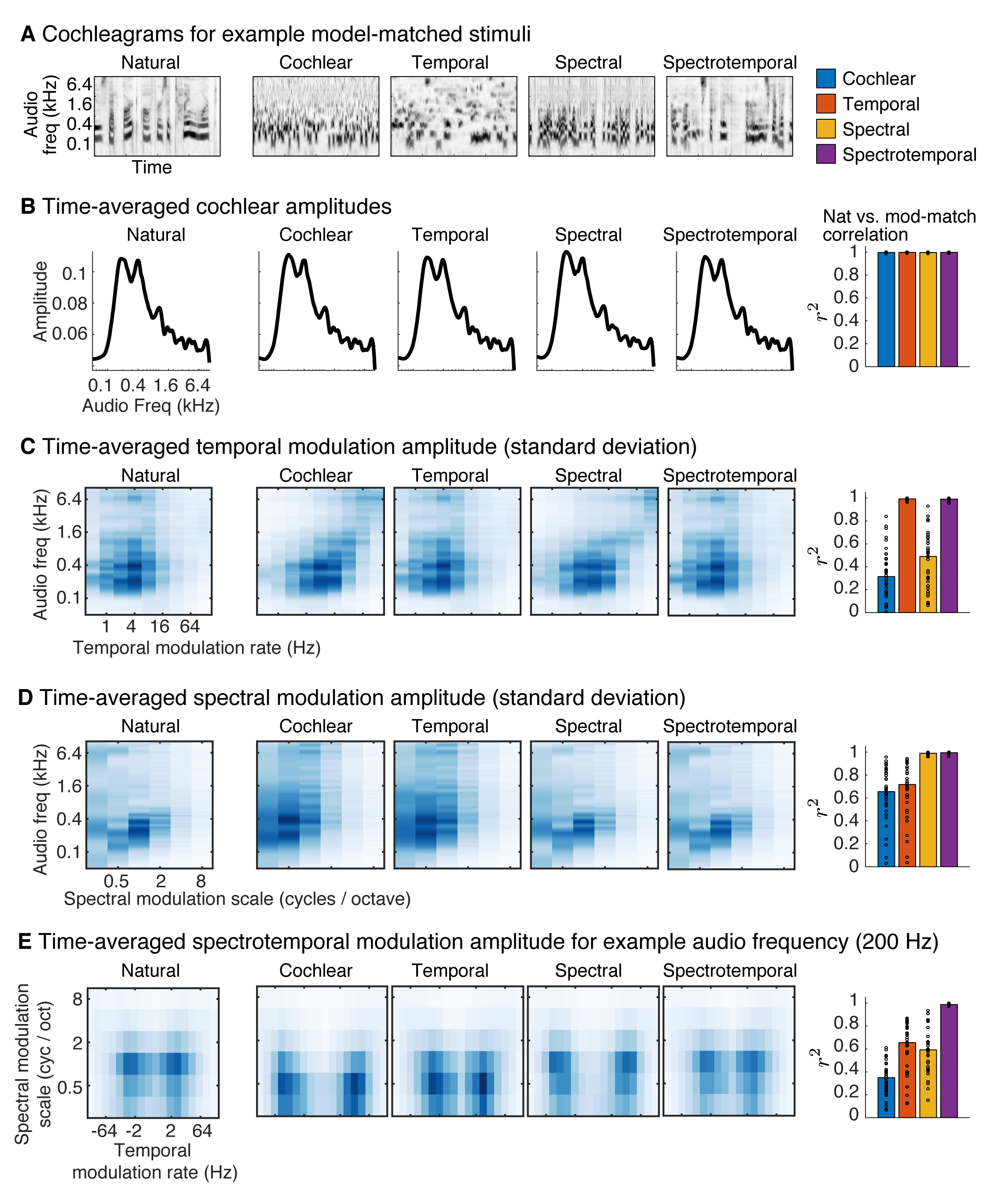

Supplement: S14 Fig — (A) Cochleagrams for a natural sound (a speech excerpt) and four corresponding model-matched sounds. (B—E) Each model was defined by a set of feature responses. Panels plot a time-averaged measure of the amplitude of each feature’s response to the example natural and model-matched sounds shown in panel A. The right-most panel plots the correlation of the filter amplitudes across all model filters for corresponding natural and model-matched sounds. Each dot corresponds to a single pair of natural/model-matched sounds. (B) Amplitude of each cochlear frequency channel envelope, averaged across time. Cochlear channel power is matched in all four conditions, as desired/expected. (C) Temporal modulation amplitude (standard deviation of each temporal modulation feature across time) for example natural and model-matched sounds. Modulation amplitude is plotted as a function of the filter’s preferred audio frequency and temporal modulation rate. (D) Spectral modulation amplitude plotted as a function of the filter’s preferred audio frequency and spectral modulation scale. (E) Spectrotemporal modulation amplitude plotted as a function of temporal modulation rate and spectral modulation scale for an example audio frequency channel (centered at 200 Hz). (TIF) [file pbio.2005127.s014.tif]

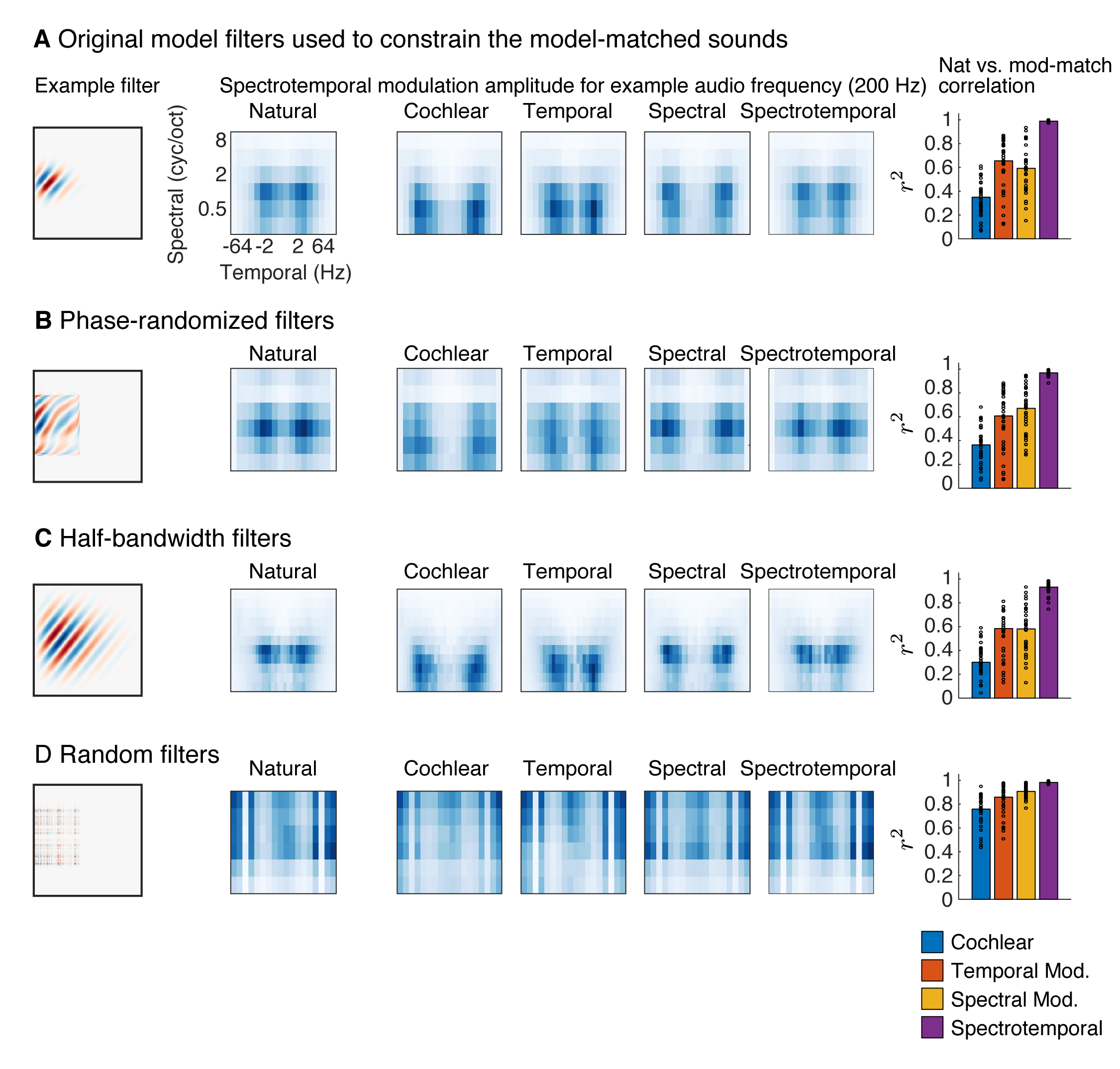

Supplement: S15 Fig — In each case, we plot an example filter from the model (left), the amplitude (standard deviation) of the filter responses as a function of the temporal rate and spectral scale for an example audio frequency channel (centered at 200 Hz) (middle), and the correlation of the amplitude across all of the filters for the natural and model-matched sounds (right) (format similar to S14E Fig). (A) The original spectrotemporal filters from Chi and colleagues (2005) that were used to constrain the model-matched sounds (same as S14E Fig). (B) Spectrotemporal filters with randomized temporal and spectral phases. (C) A model with narrower bandwidths and more filters to compensate (these filters are broader in extent when visualized in the time-frequency plane). (D) A random filter basis with variable temporal and spectral extent. In all four cases, the measured modulation power is similar for the natural and model-matched sounds. This suggests that voxels with similar responses to natural and model-matched sounds are compatible with a wide range of spectrotemporal modulation filters, and that a wide range of such filters are ruled out as descriptions of voxels that give different responses to natural and model-matched sounds, such as those we observed in nonprimary regions. (TIF) [file pbio.2005127.s015.tif]

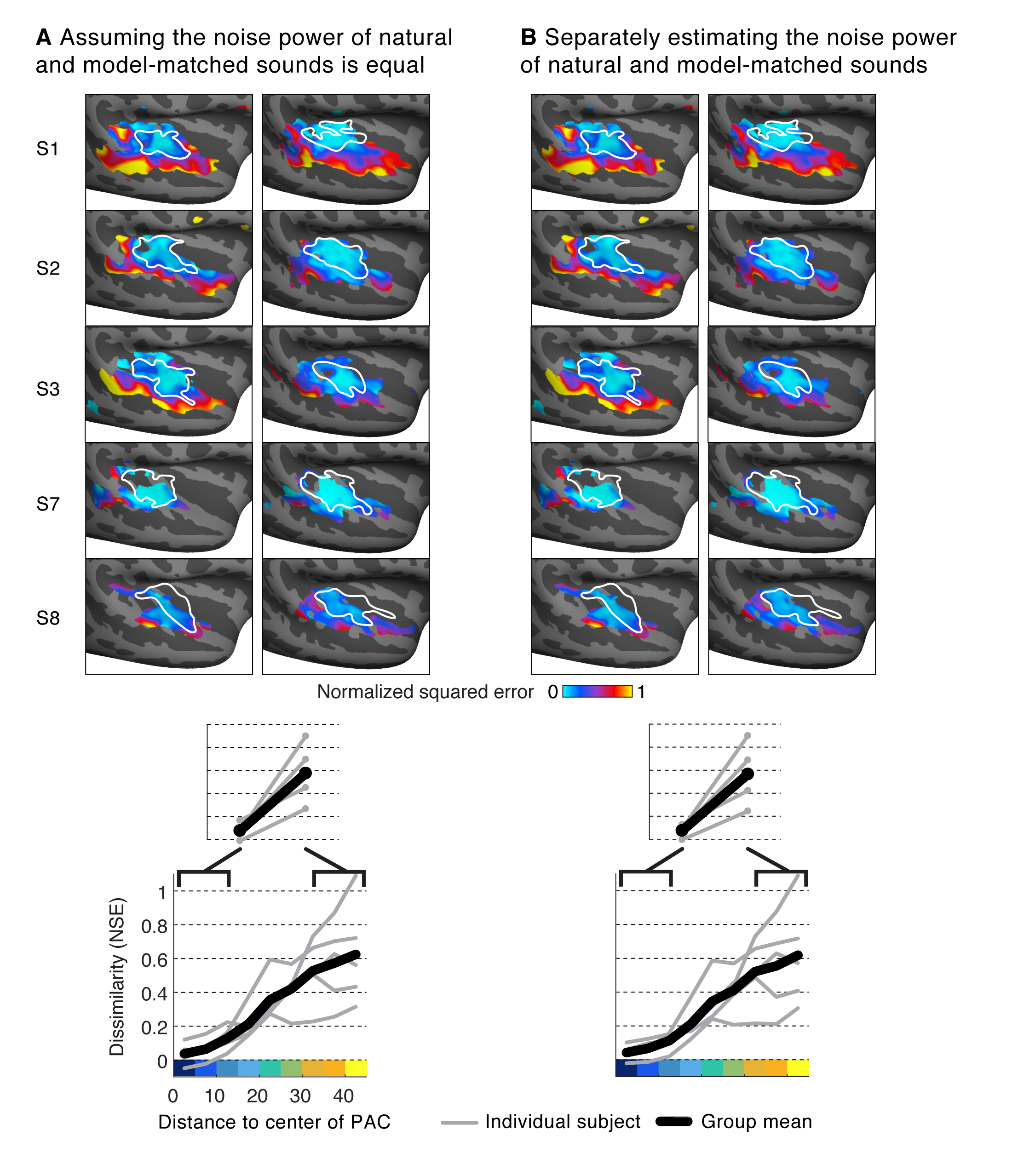

Supplement: S16 Fig — Noise-correction required estimating the power of the noise for natural and model-matched sounds. For Paradigm I, only responses to natural sounds were repeated in each scan. Using data from Paradigm II, we tested whether it is necessary to separately estimate the noise power for natural and model-matched sounds or whether one can assume they are equal. (A) Noise-corrected NSE value computed by assuming the noise power for natural and model-matched sounds is equal, using only responses to natural sounds to compute it. (B) Noise-corrected NSE values computed by separately estimating the noise power for natural and model-matched sounds (same maps as those in Fig 3C). Results are similar in both cases. NSE, normalized squared error. (TIF) [file pbio.2005127.s016.tif]

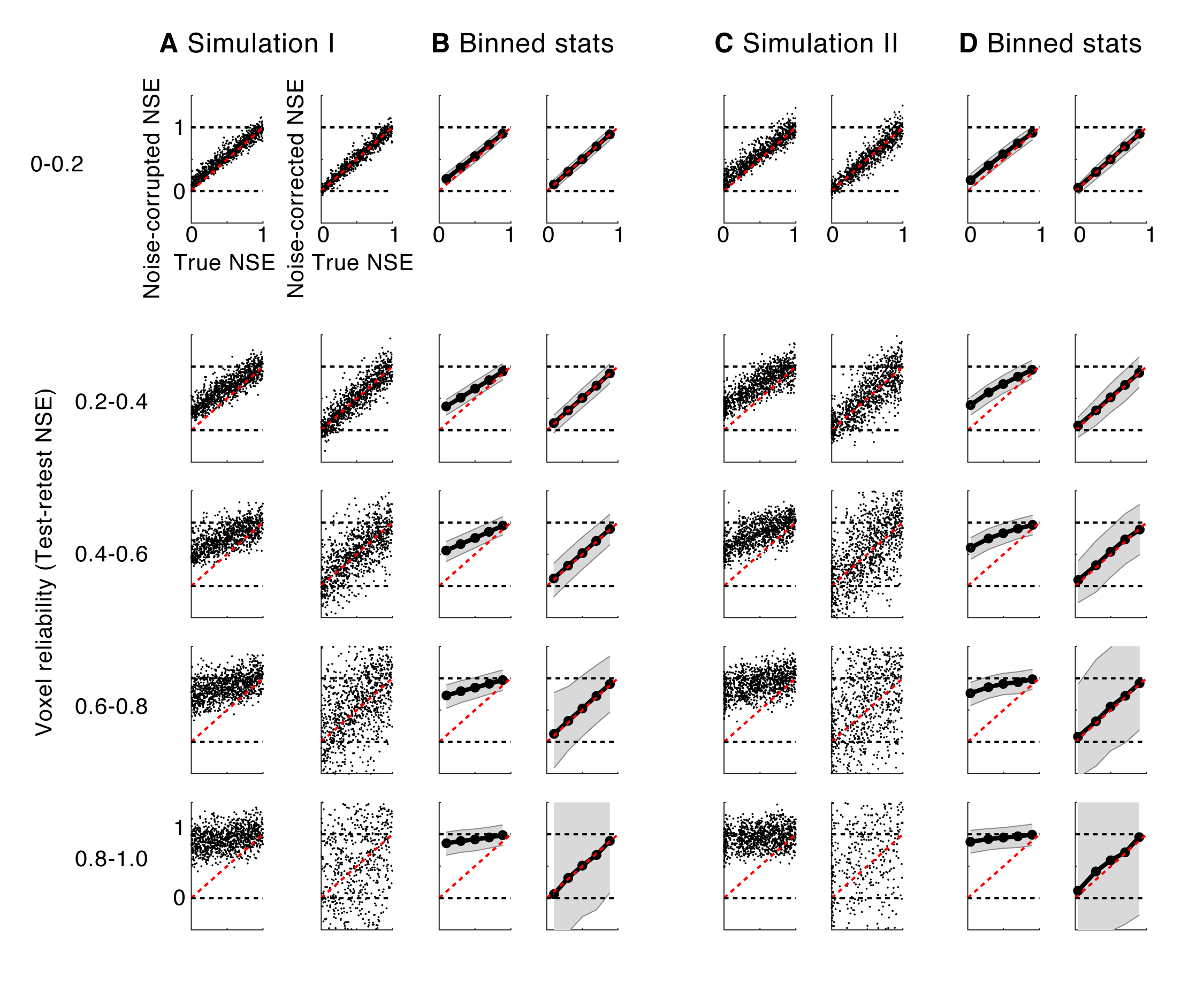

Supplement: S17 Fig — (A) Each dot corresponds to a single simulated voxel. The noise-corrupted and noise-corrected NSE values are plotted against the NSE values of the true signals uncorrupted by noise. Results have been grouped by the reliability of the simulated voxel responses, as measured by the test-retest NSE of the voxel responses (from high to low reliability, going from top to bottom). (B) The median and standard deviation (central 68% of samples) of the noise-corrupted or noise-corrected NSE values. (C–D) Same as for panels A and B, but for Simulation II (see “Evaluating the noise-corrected NSE with simulated data” in Materials and methods for details of the two simulations). NSE, normalized squared error. (TIF) [file pbio.2005127.s017.tif]

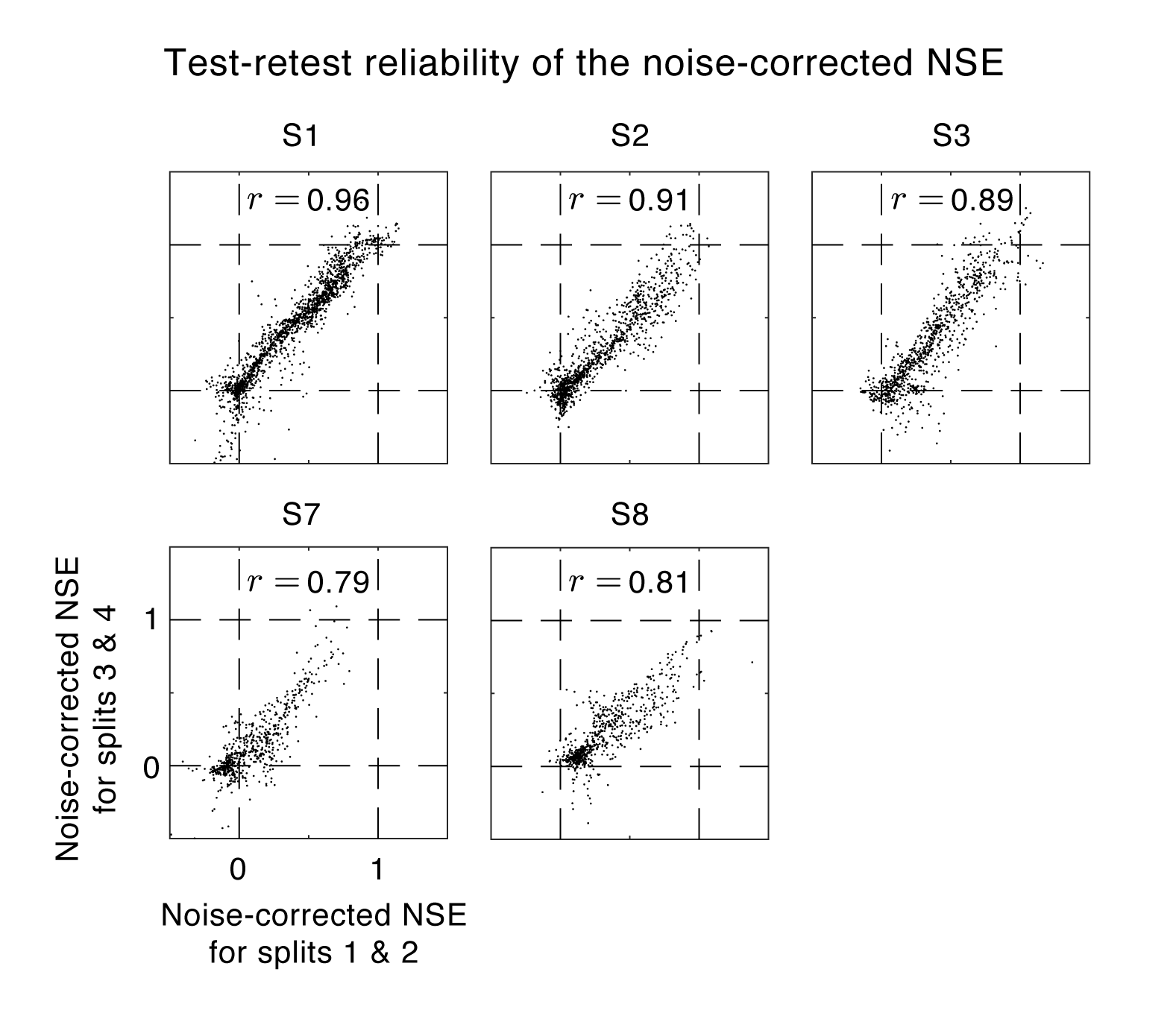

Supplement: S18 Fig — Each dot corresponds to a single voxel. The Spearman rank correlation is shown at the top of each plot for each subject. Results are shown for subjects scanned in Paradigm II, for which there was sufficient data to compute two separate estimates of the noise-corrected NSE (which requires four splits of data). NSE, normalized squared error. (TIF) [file pbio.2005127.s018.tif]
